# Implementation of universal health coverage by South Korea during the COVID-19 pandemic

**DOI:** 10.1016/j.lanwpc.2021.100093

**Published:** 2021-01-29

**Authors:** Deepa Dongarwar, Hamisu M. Salihu

**Affiliations:** a Center of Excellence in Health Equity, Training, and Research, Baylor College of Medicine, Houston, TX, United States; b Department of Family Medicine, Baylor College of Medicine, Houston, TX, United States

COVID-19 - the worst pandemic of the current century, has infected more than 80 million people, resulting in nearly two million deaths by the end of the year 2020. The pandemic has highlighted multiple issues in the existing healthcare delivery system throughout the world including racial/ethnic disparities in receipt of health care, differences in health outcomes by socio-economic and healthcare coverage status. Despite the few limitations of universal health care (UHC), including long wait times and limited usage of novel healthcare technology because of budgetary constraints [1], there are notable benefits of UHC, such as equitable health coverage for the entire population, lower cost of healthcare to society, reduced administrative costs, etc. [2]. In the beginning of the pandemic, it was observed that the case fatality rate of countries with UHC during COVID-19 was twice as that of countries without UHC (10•5% versus 4•9%, respectively) [2].

Lee et al. presented the results from their investigation of whether socio-economic disparities persisted in COVID-19 health outcomes in spite of the barrier-free health care initiative in South Korea (i.e. no out-of-pocket expense for diagnosis and treatment related to COVID-19) [3]. Almost all South Koreans (97%) are covered by the National Health Insurance Service (NHIS) and pay NHIS premiums according to their income levels or property values. The other 3%, who are Medical Aid (MA) recipients, are unable to pay premiums; and their medical costs are covered by the government. Both NHIS and MA cover health care expenditures (outpatient, inpatient, diagnostic testing fees and prescribed drugs) for an out-of-pocket cost ranging from 0 to 20%; however, due to the pandemic, the South Korean government terminated all out-of-pocket costs associated with COVID-19 related health services.

The findings reported by Lee and colleagues are consistent with other research, which showed that older individuals, males and those with comorbidities had higher prevalence and likelihood of COVID-19 infection and death [4]. In South Korea, residents of Daegu and Gyeongsangbuk provinces, and those residing in metropolitan areas exhibited greater infection and case fatality rates than individuals living elsewhere.

Compared to those on NHIS coverage, MA recipients had higher prevalence of COVID-19 (424•3 vs. 136•3 per million), a greater mortality rate (28•3 vs. 3•6 per million), as well as a higher case fatality ratio (6•7% vs. 2•7%). However, when adjusted for covariates, insurance type was not associated with case fatality. Even in areas where the health care system had collapsed, socio-economic status was not independently associated with COVID-19 case fatality [3]. A plausible explanation is that unmeasured cumulative effects of social disadvantage captured as social determinants of health (SDOH) [5] could have also influenced the results, and may need to be addressed in future studies.

At the beginning of the pandemic, South Korea was the fifth best country in the world in terms of their disaster preparedness and management protocols [6]. By following a ‘Three T Strategy’ (Testing-Tracing-Treatment) in response to COVID-19 [7], South Korea achieved a temporary, perfect UHC model in terms of the extent of health service cost coverage for patients. Having all COVID-19 diagnoses and treatments free of charge, regardless of income level, effectively supported this strategy. Moreover, by promoting transmission-reducing behaviours, including wearing a masque, washing hands, and social distancing; by improving the infrastructure, such as the availability of spare beds and community treatment centres; and by organizing a rapid response team to prevent community transmission, South Korea was able to contain the spread of the virus, thereby preventing the collapse of the healthcare system from overwhelming levels of severe cases requiring hospitalization. The country has essentially become a model setting for exploring regional preparedness and the collective impact of UHC during this pandemic [3].

Lee and colleagues’ paper [3] highlights the success story of South Korea in implementing UHC, in a very onerous circumstance within a short timeframe with the goal of achieving equity in health care delivery dispensation. Despite the efforts and laudable accomplishments, some unanswered queries remain – Will other countries with partial UHC similar to South Korea, realise comparable outcomes or are there unmeasured sources of variability that need to be accounted for including severity of clinical presentation of patients, availability of clinical and laboratory support infrastructure, optimized clinical management protocols, such as types and availability of therapeutics that could dampen disease progression and reduce case fatality?

As demonstrated by Lee et al., COVID-19 outcomes are not associated with economic status, but are more so dependent on patient demographic characteristics (e.g., age) and pre-existing comorbidities [3]. Given this premise, are there any clinical benefits to motivate implementation of UHC in countries that do not have it institutionalized at the moment, regardless of population structure and chronic disease burden as COVID-19 pandemic rages? Would a country's characteristics, such as the racial/ethnic composition, median age, immunization record, literacy, climate, disaster preparedness, type of government (a proxy for shared participatory values and population engagement) influence COVID-19 outcome trajectory with introduction of UHC?

Now, with COVID-19 vaccines progressively becoming available across the globe, it would be interesting to evaluate the distribution strategy being adopted by South Korea under the current UHC plan, and determine its impact on COVID-19 disease control and mitigation of associated fatality. An important take-home message from the article by Lee et al. is that for a durable COVID-19 pandemic control, emphasis should be on shifting our primary focus and resources more towards vaccination coverage to induce effective population herd immunity, prevention of the disease by promoting transmission-reducing behaviours, educating and training citizens and improving disaster preparedness while disease management and curative care take a secondary role.

## Author contributions

DD designed the study and takes responsibility for the integrity of the data and the accuracy of the information. HMS provided review and comments.

All authors critically revised the manuscript and approved for the version to be published.

## Declaration of Interests

None of the authors have any competing interests to report.
